# Diversity and Ecology of Marine Algicolous *Arthrinium* Species as a Source of Bioactive Natural Products

**DOI:** 10.3390/md16120508

**Published:** 2018-12-14

**Authors:** Young Mok Heo, Kyeongwon Kim, Seung Mok Ryu, Sun Lul Kwon, Min Young Park, Ji Eun Kang, Joo-Hyun Hong, Young Woon Lim, Changmu Kim, Beom Seok Kim, Dongho Lee, Jae-Jin Kim

**Affiliations:** 1 Division of Environmental Science & Ecological Engineering, College of Life Sciences & Biotechnology, Korea University, Seoul 02841, Korea; hym011@korea.ac.kr (Y.M.H.); rudndjs@korea.ac.kr (K.K.); sun-lul@korea.ac.kr (S.L.K.); dress8@korea.ac.kr (J.-H.H.); 2 Division of Biotechnology, College of Life Sciences & Biotechnology, Korea University, Seoul 02841, Korea; mogijjang@korea.ac.kr (S.M.R.); min0@korea.ac.kr (M.Y.P.); heyyo9725@korea.ac.kr (J.E.K.); biskim@korea.ac.kr (B.S.K.); 3 School of Biological Sciences and Institute of Microbiology, Seoul National University, Seoul 08826, Korea; ywlim@snu.ac.kr; 4 Microorganism Resources Division, National Institute of Biological Resources, Incheon 22689, Korea; snubull@korea.kr

**Keywords:** antioxidant, biological control, ecological role, gentisyl alcohol, multi-gene phylogeny, tyrosinase inhibition

## Abstract

In our previous study, all *Arthrinium* isolates from *Sargassum* sp. showed high bioactivities, but studies on marine *Arthrinium* spp. are insufficient. In this study, a phylogenetic analysis of 28 *Arthrinium* isolates from seaweeds and egg masses of *Arctoscopus japonicus* was conducted using internal transcribed spacers, nuclear large subunit rDNA, β-tubulin, and translation elongation factor region sequences, and their bioactivities were investigated. They were analyzed as 15 species, and 11 of them were found to be new species. Most of the extracts exhibited radical-scavenging activity, and some showed antifungal activities, tyrosinase inhibition, and quorum sensing inhibition. It was implied that marine algicolous *Arthrinium* spp. support the regulation of reactive oxygen species in symbiotic algae and protect against pathogens and bacterial biofilm formation. The antioxidant from *Arthrinium* sp. 10 KUC21332 was separated by bioassay-guided isolation and identified to be gentisyl alcohol, and the antioxidant of *Arthrinium saccharicola* KUC21221 was identical. These results demonstrate that many unexploited *Arthrinium* species still exist in marine environments and that they are a great source of bioactive compounds.

## 1. Introduction

Among a number of marine fungi isolated from a brown alga *Sargassum* sp., *Arthrinium* spp. showed high levels of cellulolytic enzyme productivity, radical-scavenging, or antifungal activities [1]. To date, however, studies on marine *Arthrinium* spp. are insufficient in terms of reliable phylogenetic analysis, physiological activity, bioactive secondary metabolites, and ecology.

The genus *Arthrinium* Kunze (sexual morph *Apiospora*; Ellis 1971 [2], Apiosporaceae) was reported as an endophyte in plant and ecologically-diverse species occurring in various habitats in both terrestrial and marine environments [3,4]. The genus *Arthrinium* has numerous broad synonyms [5]. *Cordella* is a potential synonym for *Arthrinium* and distinguished primarily by the existence of setae. *Pteroconium*, with the uncertain status of its generic name, has been regarded as an individual genus separate from *Arthrinium*, although *Apiospora* is the sexual morph of both *Pteroconium* and *Arthrinium* [2,5,6]. Currently, 80 *Arthrinium* species are reported in Index Fungorum (2018), but most *Arthrinium* taxa do not have enough sequence data and lack a detailed description of their morphological characteristics. Recently, the genus *Arthrinium* was re-examined by phylogenetic analysis of 16 species, which suggested that it is necessary to use β-tubulin (TUB) and translation elongation factor (TEF) regions to resolve species complexes [4]. Recently, Wang et al. (2018) published eight new *Arthrinium* spp. using three combined loci and multi-gene phylogenetic analysis with high bootstrap supports [7].

Marine-derived fungi have been suggested as a novel source of bioactive secondary metabolites, which can be applied in the pharmaceutical and cosmetic industry [8]. This suggestion was due to cephalosporin C production by *Acremonium chrysogenum* being reported in 1961 as the first bioactive metabolite from marine fungi, but novel natural compounds from marine fungi were less explored in comparison with compounds from terrestrial fungi [9]. Marine natural compounds have provided sufficient interest to the pharmaceutical industry due to their unique chemical properties [10]. In particular, marine fungal endophytes have been recognized as potential producers for bio-based commercial products [11]. Marine *Arthrinium* spp. have been reported as an endo-symbiont of marine algae, especially brown algae. They have been discovered to produce natural compounds such as arthrinone, arthrichitin, terpestacin, (1*R*,2*S*,3a*S*,8a*R*)-3a,6-dimethyl-1-(propan-2-yl)-1,2,3,3a,4,7,8,8a-octahydroazulene-1,2-diol (CAF-603), norlichexanthone, myrocins, libertellenones, spiroarthrinols, and griseofulvin derivatives [12,13,14,15]. However, a limited number of bioactive compounds were reported: arthpyrones F–I and apiosporamide (antibacterial) [16]; arthrinins A–D (antitumoral and antiproliferative) [13]; decarboxyhydroxycitrinone, myrocin A, libertellenone C, and cytochalasin E (antiangiogenic); arthone C and 2,3,4,6,8-pentahydroxy-1-methylxanthone (antioxidant) [17]; and cytochalasin K and 10-phenyl-[12]-cytochalasin Z_16_ (cytotoxic) [18]. In this regard, it was suggested that marine *Arthrinium* spp. have great potential to produce various bioactive compounds that can be the ingredients of a wide range of bioagents, many of which have not been discovered yet. In this study, the biological activities of marine *Arthrinium* spp. extracts were investigated to evaluate the value of marine *Arthrinium* spp. as a source of bioactive compounds and to infer their ecological role in symbiotic relationships with marine algae.

The aims of this study were (1) to identify and phylogenetically analyze marine-derived *Arthrinium* spp., (2) to evaluate the pharmaceutical value of marine *Arthrinium* spp. as a source of bioactive compounds, (3) to evaluate the ecological role in symbiosis with brown algae, and (4) to isolate and structurally identify a bioactive compound from marine *Arthrinium* species.

## 2. Results and Discussion

### 2.1. Identification and Multi-Gene Phylogeny

**Figure 1:**
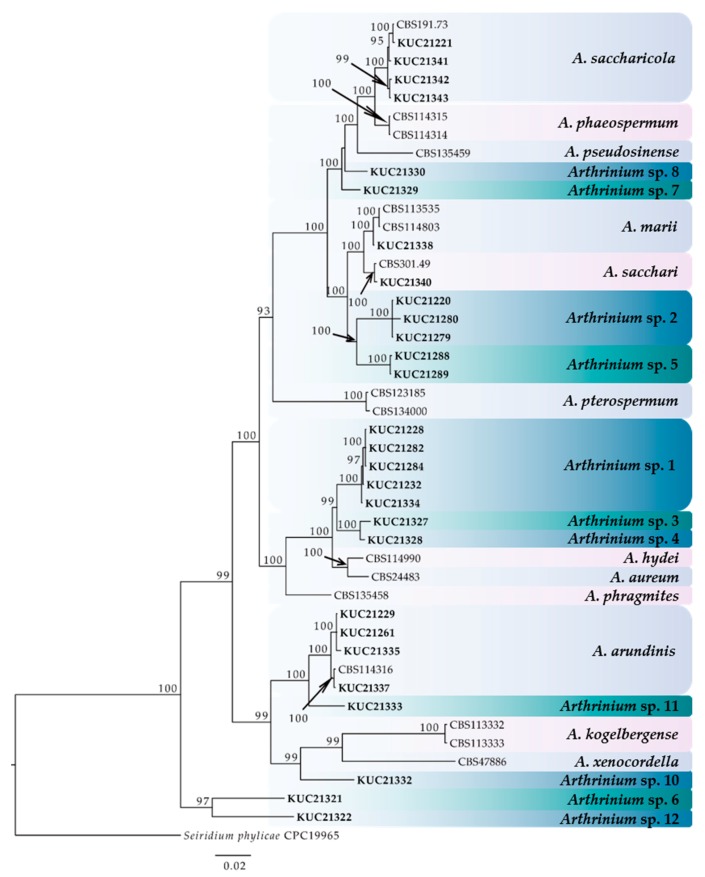
The phylogenetic tree of the *Arthrinium* species based on the combined ITS, LSU, TUB, and EF-1α sequence alignment from Bayesian analysis using MrBayes v. 3.2.1. The dataset was created from 45 taxa and 2363 characters. The fungal cultures examined in this study are boldfaced. The number above the branch indicates the posterior probability values ≥70. The scale bar indicates nucleotide substitutions per position.

A total of 28 marine-derived *Arthrinium* strains were isolated from an unknown seaweed, *Sargassum* sp., *Agarum cribrosum*, and egg masses of *Arctoscopus japonicus* (Table 1). For the phylogenetic analysis, we constructed a phylogenetic tree using combined datasets from internal transcribed spacers (ITS), partial nuclear large subunit rDNA (LSU), TUB, and TEF 1-alpha (EF-1α) region sequences containing 45 taxa and 680, 559, 590, and 534 nucleotide characters, respectively (Figure 1). As the results of the MrModeltest with the Akaike information criterion (AIC), a general time reversible (GTR) + proportion of invariable sites (I) + gamma distribution (G) model was chosen for LSU and TUB, and the symmetrical model (SYM) + I + G model and Hasegawa-Kishino-Yano (HKY) + I + G model were chosen for ITS and EF-1α, respectively. The form of the tree demonstrated that all the species were clustered with high posterior probabilities (95–100%). As a result, the 28 *Arthrinium* strains were divided into 15 species, including 11 novel species candidates. All new species will be reported in the near future.

### 2.2. Marine Habitat of Arthrinium

Most of the *Arthrinium* species isolated from each substrate were identified to be different (Table 1). Seven different *Arthrinium* species were isolated from the egg masses of *A. japonicus*. The two known species were *Arthrinium saccharicola* and *A. sacchari*, and the other five strains were considered novel *Arthrinium* candidates. Interestingly, *A. saccharicola*, *A. arundinis*, and *Arthrinium* sp. 1 were found in both *Sargassum fulvellum* and the egg masses of *A. japonicus* [19]*. A. japonicus* spawns the largest number of eggs on *Sargassum fulvellum*, one of the major endophytic hosts of *Arthrinium* spp. [20]. It was strongly speculated that the separation of the same *Arthrinium* species from both substrates was based on this fact. The conidia of the algicolous *Arthrinium* spp. can easily be transferred to the egg masses of *A. japonicus*. In another study, the fungal diversity of the *A. japonicus* egg masses were investigated, and eight *Arthrinium* species were founded: *A. saccharicola*, *A. sacchari*, *A. phaeospermum*, *A. arundinis*, and *A. rasikravindri*, as well as three unknown *Arthrinium* species [19]. According to Park et al. (2018), *Arthrinium* accounts for a small portion of the strains isolated from egg masses of *A. japonicus*. Nonetheless, the fact that there were many new species candidates suggests that various unexplored *Arthrinium* species are present in the marine environment.

*A. saccharicola*, known as a plant pathogenic fungus, has been isolated from living and dead culms of *Phragmites australis* and even from the air in the Netherlands and France and from seawater in mangrove habitats in Hong Kong [4,21]. This suggested that *A. saccharicola* has low host specificity. Additionally, *A. arundinis*, *A. marii*, and *A. sacchari* have been isolated from more than one substrate in another study [7]. *A. arundinis* has been isolated from the leaf of *Hordeum vulgare* and living leaves of *Fagus sylvatica* in Iran and Switzerland [4]. Additionally, in a recent study, 19 strains of *A. arundinis* were isolated from nine different hosts in China [7]. Taxonomical work on *Arthrinium* species isolated from marine ecosystems has been relatively lacking compared to work on those isolated from terrestrial ecosystems. Thus, more taxonomic and systematic analyses of marine-derived *Arthrinium* spp. are needed.

### 2.3. Biological Activities of Marine *Arthrinium* spp.

#### 2.3.1. Antioxidant Activity

Since *Arthrinium* spp. showed high antioxidant activity, other marine-derived *Arthrinium* species were also expected to do so [1]. The results showed that almost every *Arthrinium* strain showed high radical-scavenging activity on the assay using 2.2′-azino-bis-3-ethylbenzothiazoline-6-sulfonic acid (ABTS) radicals as a substrate (Table 1). In particular, the crude extract of *Arthrinium* sp. 3 KUC21327 exhibited even higher ABTS radical-scavenging activity than ascorbic acid. Reactive oxygen species (ROS) are responsible for many kinds of diseases and aging because they damage biomolecules, such as DNA and protein, through oxidative chain reactions [22]. This suggests that the symbiotic relationship between *Arthrinium* spp. and their major host, seaweeds, may be based on the regulation of the ROS-defense system. Many seaweeds have been used as functional food and cosmetic ingredients because of their great antioxidant ability [23,24]. It was speculated that these algicolous fungi might contribute to the antioxidant ability of seaweeds.

Researchers have discovered many kinds of antioxidants, which can scavenge free-radicals generated from ROS, and filamentous fungi were one of the main natural sources of antioxidants. Filamentous fungi produce a wide range of secondary metabolites, and many polyphenolic compounds have been reported to exhibit high radical-scavenging abilities against ROS and free radicals [25]. In the case of marine *Arthrinium* spp., arthone C and 2,3,4,6,8-pentahydroxy-1-methylxanthone were reported as antioxidants [17]. Our ABTS assay results imply that marine-derived *Arthrinium* spp. commonly produce certain kinds of antioxidant compounds. Among them, *A. sacchari* KUC21340, *A. saccharicola* KUC21221, *A. saccharicola* KUC21343, and *Arthrinium* sp. 10 KUC21332 exhibited high radical-scavenging activity against the 2,2-diphenyl-1-picrylhydrazyl (DPPH) radical. Because the reaction media of ABTS and DPPH assays differ (PBS buffer and 80% MeOH, respectively), the measured activity is affected by the solubility of potential antioxidant compounds for each solvent [26]. These four strains produce antioxidant compounds that are less hydrophilic than those of the other strains and remain active in MeOH. In particular, the IC_50_ values of *A. saccharicola* KUC21221 and *Arthrinium* sp. 10 KUC21332 were significantly lower than those of the other species, implying that these species have high antioxidative capacities. The high radical-scavenging activity of *A. saccharicola* KUC21221 extract was already reported [1]. Among the two candidates, *Arthrinium* sp. 10 was selected in consideration of the novelty of this fungal species, its antioxidant compound was separated, and the chemical structure was identified.

#### 2.3.2. Antifungal Activity

In our previous study, all six *Arthrinium* sp. isolated from *Sargassum* sp., a brown alga, exhibited high antifungal activities against terrestrial plant pathogens [1]. As *Arthrinium* spp. are one of the key endophytes of seaweed, some *Arthrinium* species were expected to have symbiotic relationships in immune mechanisms by producing antibiotics against other marine microorganisms harmful to the algae. As a result, the extracts of three *A. saccharicola* (KUC21221, KUC21341, and KUC21343) and *Arthrinium* sp. 2 KUC21220 showed inhibitory effects against mycelial growth of the test fungus *Asteromyces cruciatus*, an alginate-degrading fungus (Table S2). Alginate is a major constituent of brown algae and plays an important role in brown algae by forming the structure of the algal biomass and physically protecting algae from pathogens [27]. Some marine fungi have been reported to be able to degrade alginate: *Asteromyces cruciatus*, *Corollospora intermedia*, *Dendryphiella arenaria*, and *D*. *salina* [28]. In particular, *A*. *cruciatus* is one of the major marine colonizers ubiquitous in the marine environment [29]. This fungus can be regarded as a potentially harmful microorganism to brown algae, as it can colonize the entire algal biomass and breakdown alginate enzymatically when the defense system of algae is weakened. Decomposition of the biomaterial leads to the collapse of the defense system.

In particular, the two *A. saccharicola* (KUC21341 and KUC21342) extracts had the highest antifungal activities as they could inhibit the growth of *A. cruciatus* at a concentration of 100 µg/mL. Since they were identified as the same species, the antifungal secondary metabolites are highly likely to be identical. This result implied that the two *Arthrinium* species, especially *A. saccharicola*, may have a symbiotic relationship with brown algae that assists its defense system against other marine fungi. Furthermore, it was strongly speculated that their antifungal metabolites can affect other pathogenic fungi, considering that six marine-derived *Arthrinium* strains (*A. arundinis* KUC21229 and KUC21261, *A. saccharicola* KUC21221, *Arthrinium* sp. 1 KUC21228 and KUC21232, and *Arthrinium* sp. 2 KUC21220) showed antifungal activity against several terrestrial plant pathogenic fungi [1]. Considering that most antimicrobials have low specificity, it was thought that they might have evolved to synthesize antifungal compounds not only for themselves, but also for their symbiotic partner, marine algae. Plants are protected by cocktails of moderately-active compounds, such as essential oils, and synergy with antimicrobials lowers the minimum effective concentration of individual antimicrobial compound to prevent the emergence of resistant pathogens [30]. Similarly, it was suggested that the broad spectrum antifungal potential of these marine algicolous *Arthrinium* spp. may contribute to improving the fitness of the algal hosts by providing a broad spectrum of defense that does not trigger the emergence of resistant pathogens. It is necessary to study the interaction between antimicrobials of *Arthrinium* spp. and secondary metabolites of the algal hosts.

#### 2.3.3. Tyrosinase Inhibition Activity

A total of eight *Arthrinium* extracts exhibited inhibitory activity against tyrosinase, and *A. sacchari* KUC21340, *Arthrinium* sp. 2 KUC21279, and *Arthrinium* sp. 7 KUC21329 were the best producers of tyrosinase inhibitors (Table S2). Tyrosinase catalyzes the oxidation of not only tyrosine, but also L-3,4-dihydroxyphenylalanine (L-DOPA), a reactive intermediate compound of melanin, to dopaquinone, which eventually converts it to pheomelanin [31]. Tyrosinase inhibitors suppress the formation of melanin in human skin and prevent the darkening of the skin tone, and researchers have discovered several inhibitors of fungal origin, such as protocatechualdehyde from *Phellinus linteus*, 6-n-pentyl-α-pyrone from *Myrothecium* sp., and kojic acid, the most widely-studied tyrosinase inhibitor, from various fungi, especially *Aspergillus oryzae* [32,33,34]. These tyrosinase inhibitors have various mechanisms, and many antioxidant compounds have been reported to exhibit tyrosinase inhibition activity by suppressing melanin biosynthesis by scavenging reactive quinone products [31]. The extracts of *A. sacchari* KUC21340, *Arthrinium* sp. 2 KUC21279, and *Arthrinium* sp. 7 KUC21329 exhibited both tyrosinase inhibitory and radical-scavenging activity, and it can be interpreted that the tyrosinase inhibitors can be identical to their antioxidant compounds. This result indicates that the three *Arthrinium* species can produce tyrosinase inhibitors with antioxidant activity that regulates melanin biosynthesis by scavenging reactive quinone products.

#### 2.3.4. Quorum Sensing Inhibition Activity

**Figure 2:**
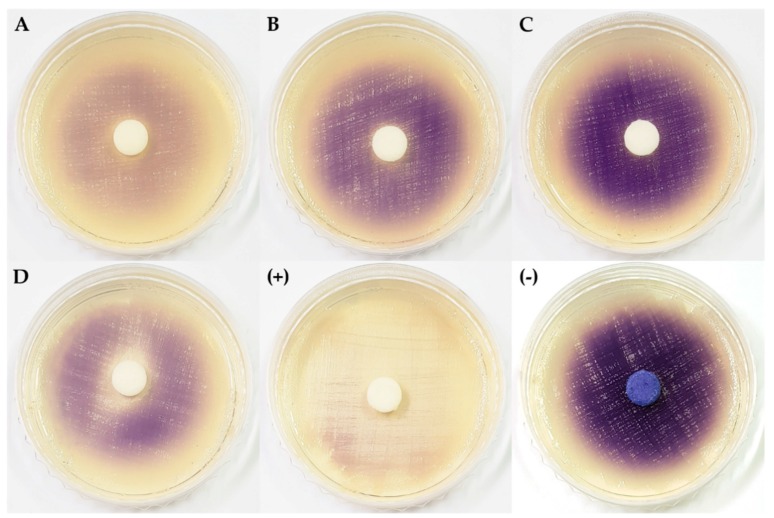
Quorum sensing inhibitory activity of extracts from four marine algicolous *Arthrinium* strains. (**A**) *Arthrinium* sp. 1 KUC21228; (**B**) *Arthrinium* sp. 1 KUC21232; (**C**) *Arthrinium* sp. 5 KUC21289; (**D**) *Arthrinium* sp. 6 KUC21321; (+) positive control (piericidin A); (−) negative control (DMSO).

The extracts of *Arthrinium* sp. 1 KUC21228 and KUC21232 and *Arthrinium* sp. 6 KUC21321 inhibited the production of violacein by *C. violaceum* CV026 in the presence of *N*-(3-oxo-hexanoyl)-l-homoserine lactone (3-oxo-C6-HSL), an *N*-acyl homoserine lactone (AHL), which is one of the most widely-studied quorum sensing (QS) molecules (Figure 2). In particular, *Arthrinium* sp. 1 KUC21228 extract showed a high inhibitory activity even comparable to that of the positive control, which is a pure compound (Figure 2A). QS is responsible for the formation of bacterial biofilms and the expression of virulence genes, and bacterial biofilms have been reported to be approximately 1000-times more resistant to antibiotics than their planktonic counterparts [35]. Some fungi produce quorum-quenching compounds such as patulin and penicillic acid from *Penicillium* spp., which can suppress bacterial biofilm formation [36]. All four *Arthrinium* strains that showed QS inhibition activity were isolated from seaweeds that are well known to produce various quorum sensing inhibitors (QSIs), namely bromoperoxidase from a brown alga *Laminaria digitate* and floridoside, bentonicine, and isoethionic acid from a Korean red alga *Ahnfeltiopsis flabelliformis* [37,38].

In fact, many filamentous fungi associated with the plant rhizosphere have been reported to degrade 3-oxo-C6-HSL enzymatically [39]. Therefore, it was suggested that these seaweed-endophytic *Arthrinium* species can produce QSIs that are expected to be lactonase or other kinds of AHL-degrading enzymes, which degrade 3-oxo-C6-HSL and assist in resistance to bacterial biofilms in seaweeds. Otherwise, it could be norlichexanthone, a known metabolite of *Arthrinium* sp., reported to inhibit the QS mechanism in *Staphylococcus aureus*, a human pathogen [40]. Considering that arthpyrones F-I and apiosporamide were reported as antibacterial compounds from marine *Arthrinium* sp., some marine *Arthrinium* species may directly inhibit seaweed-pathogenic bacteria [16]. The three new seaweed-endophytic *Arthrinium* strains were revealed to be potential QSI producers, which makes them valuable as a source of biological compounds that inhibit the bacterial biofilm, which causes huge economic losses in many areas, such as food, aquaculture, wastewater treatment, and the shipping industry [41,42]. The selected fungi will be subjected to an in vitro experiment as a subsequent screening by quantifying their activity.

### 2.4. Antioxidant Compound from *Arthrinium* sp. 10

**Figure 3:**
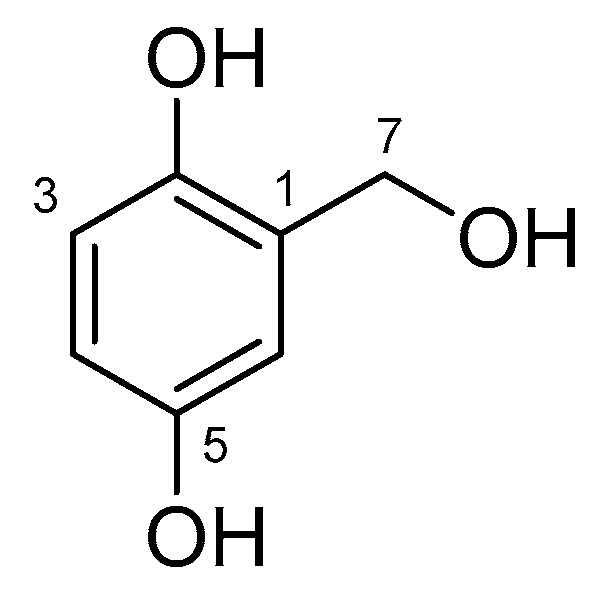
Structure of Compound **1**.

The extract of *Arthrinium* sp. 10 KUC21332, a new species candidate with the highest radical-scavenging activity, was selected to identify the active compound. A bioassay-guided isolation of this extract afforded gentisyl alcohol (**1**) (Figure 3). The chemical structure was identified using spectroscopic data and a comparison with literature data [43]. The ^1^H and ^13^C NMR spectra, LC chromatogram, UV spectrum, and MS spectra of Compound **1** are shown in the Supplementary Materials (Figures S1–S4). In addition, the results of comparative analysis of *A. saccharicola* KUC21221 extract, *Arthrinium* sp. 10 KUC21332 extract, and gentisyl alcohol using ultra-performance liquid chromatography (UPLC) with a photodiode array (PDA) detector confirmed that the antioxidant compound of *A. saccharicola* KUC21221 is the same as that of *Arthrinium* sp. 10 KUC21332, that is gentisyl alcohol (Figure S5).

The radical-scavenging activity of Compound **1** was evaluated using the ABTS and DPPH assays. As described in Table 2, it exhibited higher activities than the positive control (ascorbic acid), which corresponds to the result of a previous report [44]. Gentisyl alcohol has also been reported to have proangiogenic, caspase inhibition, histone deacetylases inhibition, antibacterial (against methicillin-resistant *Staphylococcus aureus*), antifungal (against *Colletotrichum gloeosporioides*), antileishmanial (against *Leishmania donovani*), and cytotoxic (against human breast and colon cancer cell lines, MCF-7 and HT-29, respectively, and against sea urchin *Strongylocentrotus intermedius*) activity [45,46,47,48,49,50,51,52,53]. This is the first report of the production of gentisyl alcohol from the genus *Arthrinium*.

## 3. Materials and Methods

### 3.1. Microorganisms

#### 3.1.1. Fungal Resources

Marine-derived *Arthrinium* spp. used in this study were obtained from the Korea University Culture (KUC) collection and Marine Fungal Resource Bank (MFRB) at Seoul National University as a marine bioresource bank of Korea by the Ministry of Oceans and Fisheries. *Asteromyces cruciatus* SFC20161110-M19 was obtained from MFRB to use as a target fungus of the antifungal assay.

The algicolous fungi were isolated according to the following procedure [1,19]. Marine algae and the egg masses of *A. japonicus* were washed with sterile 3.44% artificial sea water (ASW) and cut into 5-mm^2^ squares. The pieces were placed on each of three culture plates: dichloran rose bengal chloramphenicol agar (Difco, Sparks, MD, USA), glucose yeast extract agar (1 g/L glucose, 0.1 g/L yeast extract, 0.5 g/L peptone, and 15 g/L agar), and Sabouraud dextrose agar (Difco, Sparks, MD, USA) supplemented with 3.44% ASW, 0.01% streptomycin, and 0.01% ampicillin to prevent bacterial growth. The plates were incubated at 25 °C for 7–15 days, and the grown fungi were transferred to a potato dextrose agar (PDA, Difco, Sparks, MD, USA) plate with 3.44% ASW periodically.

#### 3.1.2. DNA Extraction, PCR, and Identification

Genomic DNA extraction was performed using fungal culture grown on malt extract agar by the AccuPrep Genomic DNA Extraction kit (Bioneer, Seoul, Korea). The DNA sequences of KUC21221, KUC21229, KUC21261, KUC21228, KUC21232, and KUC21220 were obtained from our previous research [1]. PCR reactions were performed with three different primer regions using the AccuPower PCR Premix Kit (Bioneer, Seoul, Korea). ITS (forward: ITS1F (5′-CTTGGTCATTTAGAGGAAGTAA-3′) and reverse: LR3 (5′-CCGTGTTTCAAGACGGG-3′)) [54,55], LSU (forward: LR0R (5′-ACCCGCTGAACTTAAGC-3′) and reverse: LR5 (5′-TCCTGAGGGAAACTTCG-3′) or LR7 (5′-TACTACCACCAAGATCT-3′)) [56], TUB (forward: T10 (5′-CATCGAGAAGTTCGAGAAGG-3′) or Bt2a (5′-GGTAACCAAATCGGTGCTGCTTTC-3′) and reverse: T2 (5′-TAGTGACCCTTGGCCCAGTTG-3′) or Bt2b (5′-ACCCTCAGTGTAGTGACCCTTGGC-3′)) [57,58], and EF-1α (forward: EF1-728F (5′-GGA(G/A)GTACCAGT(G/C)ATCATGTT-3′) and reverse: EF2 (5′-GGA(G/A)GTACCAGT(G/C)ATCATGTT-3′)) [59,60] were amplified under the temperature cycling parameters as follows. For ITS and LSU, 95 °C for 4 min, followed by 30 cycles of 95 °C for 30 s, 55 °C for 30 s, and 72 °C for 30 s; an elongation step of 72 °C for 5 min was performed at the end. For TUB, 95 °C for 5 min, followed by 30 cycles of 95 °C for 35 s, 55 or 56 °C for 50 s, and 72 °C for 2 min; an elongation step was performed at 72 °C for 7 min. For EF-1α, 94 °C for 2 min, followed by 29 cycles of 93 °C for 30 s, 55 or 56 °C for 30 s, and 72 °C for 1 minute; an elongation step was performed at 72 °C for 10 min. DNA sequencing was executed by Macrogen (Seoul, Korea) using the Sanger method with a 3730xl DNA Analyzer (Life technology, Carlsbad, CA, USA). The obtained DNA sequences were submitted to the GenBank, and the accession numbers are presented in Table 1.

#### 3.1.3. Phylogenetic Analysis

The obtained DNA sequences with reference sequences (Table S1) obtained from GenBank using a BLAST search were proofread, aligned using MAFFT 7.397, and modified manually using MacClade v4.08 (http://macclade.org) [61,62]. They were tested by MrModeltest v2.3 (https://www.softpedia.com/get/Science-CAD/MrModeltest.shtml) with default options using the AIC [63]. Bayesian analysis was performed by MrBayes v3.2.1 (http://nbisweden.github.io/MrBayes) [64]. Two operations were performed and contained 1,000,000 generations. For each operation, the result of every 100th generation was sampled. Among them, the first 25% of the trees was removed, and the last 75% was selected. A phylogenetic tree was constructed according to the 50% majority-rule, and tree reliability was confirmed by the posterior probability.

### 3.2. Preparation of Fungal Extracts

All of the fungal species were precultured on 20-mL Petri dishes containing PDA at 25 °C until mycelia covered the dishes. After the preculture period, three agar plugs with mycelia were transferred to Petri dishes (150 × 20 mm) containing 50 mL of PDA and incubated at 25 °C for seven days in darkness. The fungal cultures were incubated in triplicate. To obtain fungal extracts, the solid media with mycelia were extracted with 200 mL of methanol for 24 h, followed by filtration with Whatman No.1 filter paper. Filtrates were dried at 37 °C under a vacuum and cooled at 4 °C during circulation. The dried residues were redissolved in a half and half percentage of water-ethyl acetate solution. After six hours, the portion of ethyl acetate was collected and evaporated under the same temperature condition mentioned above. The dried extracts were maintained in the 20-mL scintillation vials at 4 °C until use. To obtain a large amount of crude extracts for separating the bioactive compound, a culture and extraction were carried out using the same method in 200 Petri dishes.

### 3.3. Biological Assays

#### 3.3.1. Antioxidant Assay

##### ABTS Radical-Scavenging Assay

ABTS (Sigma-Aldrich, Inc., St. Louis, MO, USA) was dissolved in phosphate-buffered saline (PBS, pH 7.4) to 7 mM. Then, the ABTS solution was mixed with potassium persulfate solution that was dissolved in PBS to 2.45 mM. The mixture was stored in the dark at room temperature for 24 h. After the ABTS^•+^ radical was formed, the ABTS solution was diluted with PBS to an absorbance of 0.70 (±0.02) at a wavelength of 734 nm. Then, 990 µL of ABTS radical solution and 10 µL of fungal extract (10 mg/mL in DMSO) were reacted in a cuvette. The absorbance was measured at 734 nm after 6 min using a spectrophotometer (Sunrise^TM^, Tecan, Männedorf, Switzerland).

##### DPPH Radical-Scavenging Assay

DPPH (Sigma-Aldrich Inc., St. Louis, MO, USA) was dissolved in 80% methanol at 150 µM. The 200 µL of DPPH solution and 22 µL of the fungal extracts (10 mg/mL) was mixed in each well in a 96-well plate. The plate was allowed to reach a steady state for 30 min at room temperature. The absorbance was measured at 540 nm.

#### 3.3.2. Antifungal Assay

Antifungal activity was determined in a 96-well plate using the microtiter broth dilution method [65]. Twenty-five microliters of spore suspensions (4 × 10^5^ conidia/mL) of *Asteromyces cruciatus* SFC20161110-M19 were added to each well containing 25 µL of 4× potato dextrose broth and 49 µL of D.W. Finally, the fungal extracts were added to a final concentration of 100 µg/mL. The inoculated 96-well plates were incubated at 25 °C, for three days. To determine the minimum inhibitory concentration (MIC), the extracts with lower concentrations (50, 25, 12.5, 6.5 µg/mL) were tested.

#### 3.3.3. Tyrosinase Inhibition Assay

A tyrosinase inhibition assay was conducted based on the method described by Lai et al. (2009) [66]. Forty microliters of fungal extract (2.5 mg/mL in 50% DMSO), 70 μL of 0.1 M potassium phosphate buffer (pH 6.8), and 30 μL of tyrosinase from mushroom (0.02 mg/mL in the buffer; Sigma Aldrich, St. Louis, MO, USA) were mixed in a 96-well plate. The mixture was heated to 30 °C for five minute, and 100 μL of 2.5 mM L-DOPA (Sigma Aldrich, St. Louis, MO, USA) was added to the well. After 30 min, the reaction was terminated by putting the plate in ice, and the absorbance was measured at 492 nm. Kojic acid (Sigma Aldrich, St. Louis, MO, USA) was used as a positive control, and a mixture without L-DOPA was regarded as a blank.

#### 3.3.4. Quorum Sensing Inhibition Assay

QSI screening was performed based on the inhibition of violacein production by *Chromobacterium violaceum* CV026 strains under culture conditions supplemented with an exogenous QS molecule, 3-oxo-C6-HSL [67]. The *C. violaceum* CV026 cultured overnight was diluted with LB medium (5 g of yeast extract, 10 g of tryptone, and 5 g of NaCl in a liter of D.W.) to an OD_600nm_ of 0.1, and 97 µL of the diluted culture were added to each well of a 96-well plate. One microliter of 3-oxo-C6-HSL (final concentration of 10 µM; Sigma-Aldrich, St. Louis, MO, USA) and two microliters of fungal extract (diluted to 10 mg/mL in DMSO) was added to each well. As a negative control and positive control, DMSO and 100 nM (*Z*)-4-bromo-5-(bromomethylene)-2(5*H*)-furanone (Furanone C-30; Sigma-Aldrich, St. Louis, MO, USA) were used, respectively. After 16 h at 28 °C, 100 µL of DMSO was added to each well and vigorously shaken for an hour at room temperature to determine the production of violacein.

The selected extracts were further examined using a paper disc method. Fifteen microliters of the diluted culture of *C. violaceum* CV026 was spread on an LB agar plate, and a paper disc (diameter 8 mm; Advantec, Tokyo, Japan) impregnated with 40 μL of extract and 1 μL of 1 mM 3-oxo-C6-HSL was placed in the center of the plate. DMSO was used as a negative control, and piericidin A isolated from *Streptomyces xanthocidicus* KPP01532 was used as a positive control [67]. The plates were incubated overnight at 28 °C.

### 3.4. Isolation and Identification of the Bioactive Compound

#### 3.4.1. Experimental Procedures

Column chromatography was performed by silica gel (200–300 mesh, Merck, Darmstadt, Germany), and prep-HPLC was performed by a Waters system consisting of a 515 pump and 2996 PDA detector (Milford, MA, USA). NMR spectra were recorded on a Varian 500-MHz NMR spectrometer (Palo Alto, CA, USA), and ESIMS spectra were recorded on an LCQ Fleet Ion Trap MS spectrometer (Thermo Scientific, Madison, WI, USA). UPLC was performed using Waters ACQUITY UPLC^TM^.

#### 3.4.2. Bioassay-Guided Isolation

The crude extract (826.2 mg) was fractionated on a column (3 × 30 cm) packed with a silica gel using elution with *n*-hexane-EtOAc gradient ratios (1:0 to 0:1) to provide seven fractions (F1–F7). Among the seven fractions, F5 exhibited 100% DPPH radical-scavenging activity at a concentration of 500 μg/mL and was separated into five peak fractions (F5-P1–F5-P5) by prep-HPLC (5–30% ACN, 8 mL/min). The first subfraction (F5-P1) exhibited 100% DPPH radical-scavenging activity at a concentration of 10 μg/mL and was further purified by silica gel column chromatography (1 × 20 cm) using elution with CHCl_3_-MeOH in gradient ratios (50:1–20:1) to provide target Compound **1** (F5-P1-2, 61.9 mg).

Gentisyl alcohol (**1**) ^1^H NMR (DMSO-*d*_6_, 500 MHz) δ_H_ 8.58 (2H, br s, overlap, OH-2 and OH-5), 6.75 (1H, d, *J* = 3.0 Hz, H-6), 6.55 (1H, d, *J* = 8.5 Hz, H-3), 6.43 (1H, d, *J* = 8.5, 3.1 Hz, H-4), 4.92 (1H, br s, OH-7), 4.41 (2H, br s, H-7); ^13^C NMR (DMSO-*d*_6_, 125 MHz) δ_C_ 150.2 (C-5), 146.8 (C-2), 129.8 (C-1), 115.5 (C-3), 114.4 (C-6), 113.7 (C-4), 58.7 (C-7); ESIMS (*m*/*z*) 139.2 [M − H]^−^, 185.1 [M + COOH]^−^.

## 4. Conclusions

The multi-gene phylogenetic analysis revealed that 28 *Arthrinium* strains isolated from various marine environments consisted of four known species and 11 new species candidates. Most of them exhibited strong antioxidant activity, and some showed antifungal, quorum sensing inhibition, and tyrosinase inhibition activity. It was demonstrated that marine algicolous *Arthrinium* spp. can support the regulation of reactive oxygen species in symbiotic algae and protect against pathogens and bacterial biofilm formation. They can form a symbiotic relationship in a way that increases the viability and environmental competitiveness of symbiotic algae in exchange for nutrients such as mannitol, which is the main photosynthetic product of brown algae [68]. A strong antioxidant compound was isolated from a new species, *Arthrinium* sp. 10 KUC21332, and identified to be gentisyl alcohol (**1**), and the antioxidant compound of *A. saccharicola* KUC21221 was identical. These results suggest that many *Arthrinium* spp. remain unexploited in the marine environment, and marine *Arthrinium* spp. are a great source of bioactive compounds. Other bioactive compounds from marine *Arthrinium* spp., including antifungal compounds, tyrosinase inhibitors, and QSIs, will be isolated and identified in the near future.

## Figures and Tables

**Table 1:** General information for the 28 marine-derived *Arthrinium* spp. and antioxidant activity of the fungal extracts.

Fungal Identity	Strain ID	Isolation Source	GenBank Accession Number				Antioxidant Activity (IC_50_, μg/mL)	
			ITS	LSU	TUB	EF-1α	ABTS ^a^	DPPH ^b^
*A. arundinis*	KUC21229	*Sargassum fulvellum*	KT207747	MH498470	MH498512	MH544684	40.55	>1000
	KUC21261	*Sargassum fulvellum*	KT207779	MH498469	MH498511	MH544683	52.33	>1000
	KUC21335	Unknown seaweed	MH498552	N.D.	MH498510	N.D.	64.89	N.D.
	KUC21337	Beach sand	MH498551	MH544659	MH498509	MH544682	>100	>1000
*A. marii*	KUC21338	Unknown seaweed	MH498549	MH498467	MH498507	MH544681	74.77	N.D.
*A. sacchari*	KUC21340	Egg masses of *Arctoscopus japonicus*	MH498548	MH498466	MH498506	MH544680	17.45	601.34
*A. saccharicola*	KUC21221	*Sargassum fulvellum*	KT207737	KT207687	KT207637	MH544679	14.09	59.19
	KUC21341	Egg masses of *Arctoscopus japonicus*	MH498547	MH498465	MH498505	N.D.	>100	N.D.
	KUC21342	Egg masses of *Arctoscopus japonicus*	MH498546	MH498464	MH498504	N.D.	58.27	N.D.
	KUC21343	Egg masses of *Arctoscopus japonicus*	MH498545	MH498463	MH498503	MH544678	38.81	744.32
*Arthrinium* sp. 1	KUC21228	*Sargassum fulvellum*	KT207746	KT207696	KT207644	MH544677	55.29	N.D.
	KUC21232	*Sargassum fulvellum*	KT207750	KT207700	KT207648	MH544676	>100	>1000
	KUC21282	*Sargassum fulvellum*	MH498544	MH498462	MH498502	MH544675	45.37	>1000
	KUC21284	*Sargassum fulvellum*	MF615228	MF615215	MF615233	MH544674	100.04	>1000
	KUC21334	Egg masses of *Arctoscopus japonicus*	MH498543	MH544661	MH498501	MH544673	50.89	>1000
*Arthrinium* sp. 2	KUC21220	*Sargassum fulvellum*	KT207736	KT207786	KT207636	MH544672	23.23	>1000
	KUC21279	*Sargassum fulvellum*	MF615229	MF615216	MF615234	MH544671	41.71	>1000
	KUC21280	*Sargassum fulvellum*	MH498542	MH544660	MH498500	N.D.	61.43	>1000
*Arthrinium* sp. 3	KUC21327	Egg masses of *Arctoscopus japonicus*	MH498541	MH498461	MH498499	MH544670	10.32	>1000
*Arthrinium* sp. 4	KUC21328	Unknown seaweed	MH498538	MH498458	MH498496	MH544669	19.22	>1000
*Arthrinium* sp. 5	KUC21288	Unknown seaweed	MF615230	MF615217	MF615235	MH544668	50.32	>1000
	KUC21289	Unknown seaweed	MF615226	MF615213	MF615231	MH544668	61.63	>1000
*Arthrinium* sp. 6	KUC21321	Unknown seaweed	MH498533	MH498453	MH498491	N.D.	46.98	>1000
*Arthrinium* sp. 7	KUC21329	Egg masses of *Arctoscopus japonicus*	MH498531	MH498451	MH498489	MH544666	39.05	>1000
*Arthrinium* sp. 8	KUC21330	Egg masses of *Arctoscopus japonicus*	MH498530	MH498450	MH498488	MH544665	72.56	> 1000
*Arthrinium* sp. 10	KUC21332	Egg masses of *Arctoscopus japonicus*	MH498524	MH498444	MH498482	MH544664	14.87	73.22
*Arthrinium* sp. 11	KUC21333	*Agarum cribrosum*	MH498520	MH498440	MH498478	MH544663	32.62	>1000
*Arthrinium* sp. 12	KUC21322	Unknown seaweed	MH498515	MH498435	MH498473	MH544662	>100	>1000
Ascorbic acid *							13.70	6.80

N.D. means no data or not detected. ^a^ ABTS, 2.2′-azino-bis-3-ethylbenzothiazoline-6-sulfonic acid; ^b^ DPPH, 2,2-diphenyl-1-picrylhydrazyl; * positive control for antioxidant activity.

**Table 2:** ABTS and DPPH radical-scavenging activity of Compound **1** isolated from *Arthrinium* sp. 10 KUC21332.

Compound	Antioxidant Activity (IC_50_, μM)	
	ABTS ^a^	DPPH ^b^
Gentisyl alcohol (**1**)	26.43	28.74
Ascorbic acid *	77.79	38.61

^a^ ABTS, 2.2′-azino-bis-3-ethylbenzothiazoline-6-sulfonic acid; ^b^ DPPH, 2,2-diphenyl-1-picrylhydrazyl; * positive control.

## Supplementary Materials

The following are available online at http://www.mdpi.com/1660-3397/16/12/508/s1: Figure S1: ^1^H NMR spectrum of Compound **1**, Figure S2: ^13^C NMR spectrum of Compound **1**, Figure S3: LC chromatogram and UV spectrum of Compound **1**, Figure S4: MS spectra of Compound **1**, Figure S5: UPLC peaks and UV spectrum of *A. saccharicola* KUC21221 extract, *Arthrinium* sp. 10 KUC21332 extract, and Compound **1**. The arrows indicate gentisyl alcohol, Table S1: GenBank accession numbers of the reference sequences used in the phylogenetic analysis in this study, Table S2: Marine algicolous *Arthrinium* spp. exhibiting antifungal and tyrosinase inhibition activity.
