# Supplementary material for: Diversity and Ecology of Marine Algicolous Arthrinium Species as a Source of Bioactive Natural Products
Source: Mar Drugs. 2018 Dec 14;16(12):508. doi: 10.3390/md16120508 (PMC6315899; doi:10.3390/md16120508)
Supplement: Supplementary file 1 [file marinedrugs-16-00508-s001.pdf]

Supplementary materials

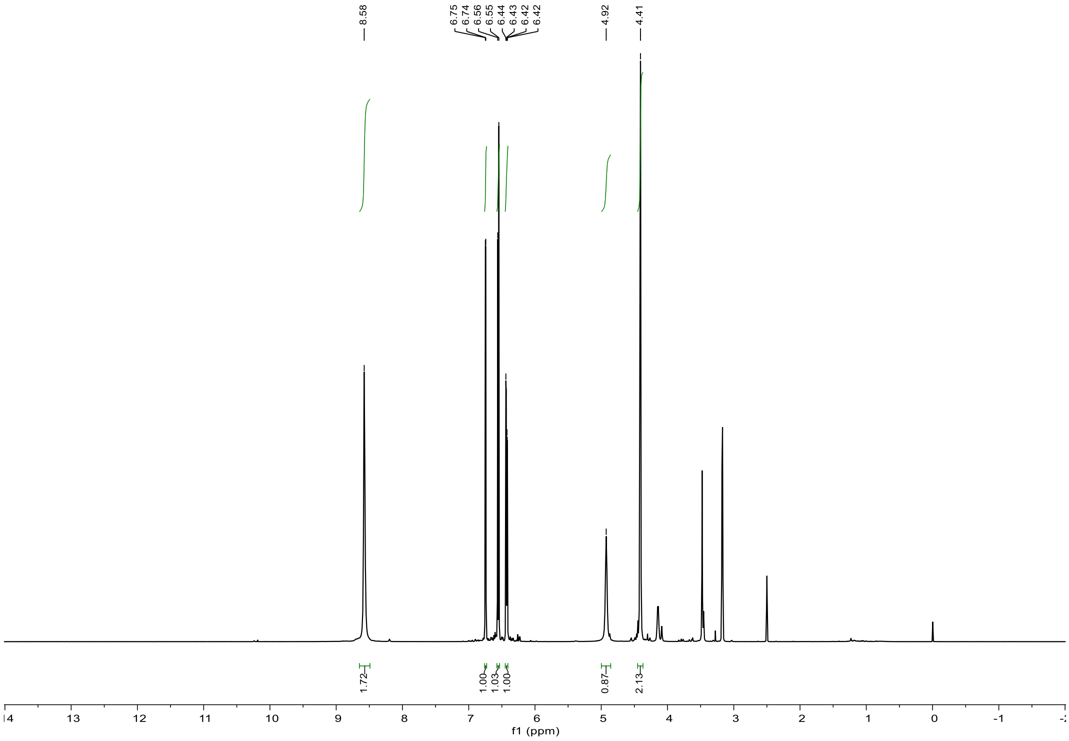

Figure S1. ^{1}H NMR spectrum of Compound **1**

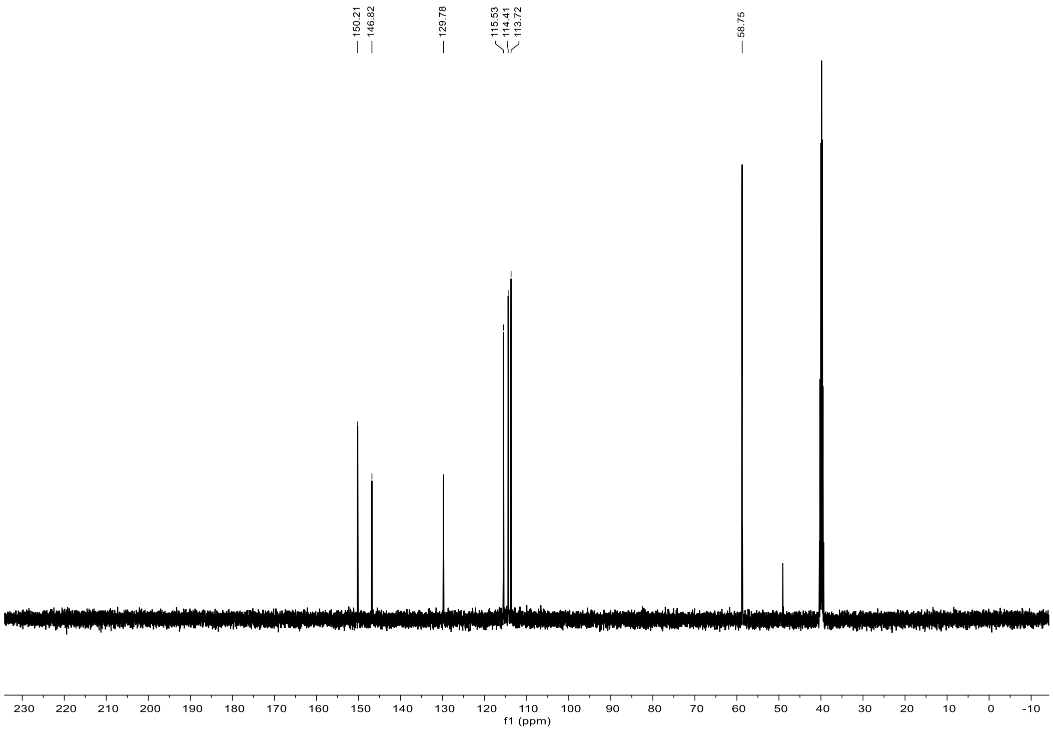

Figure S2. ^{13}C NMR spectrum of Compound **1**

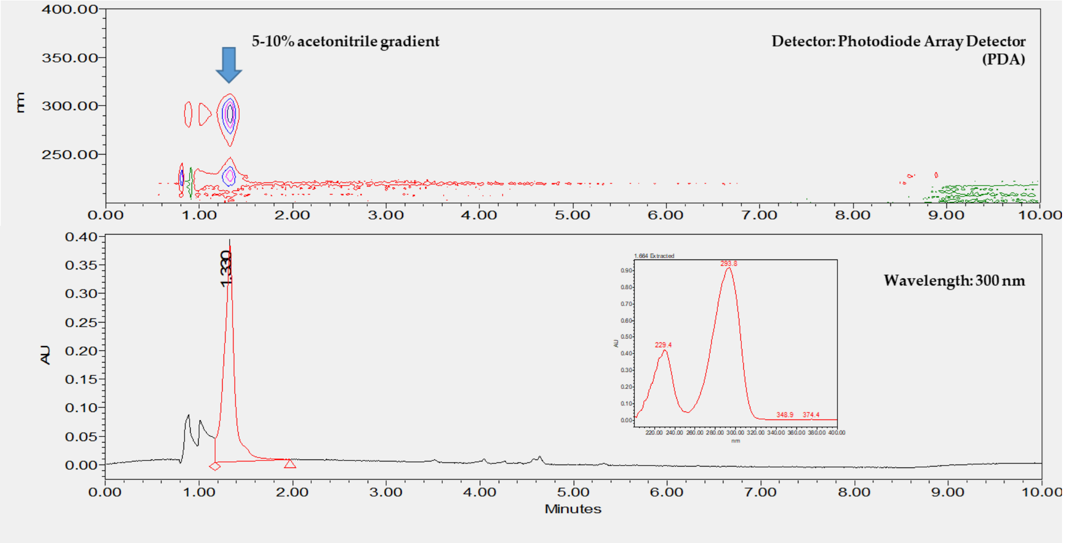

Figure S3. LC chromatogram and UV spectrum of Compound **1**

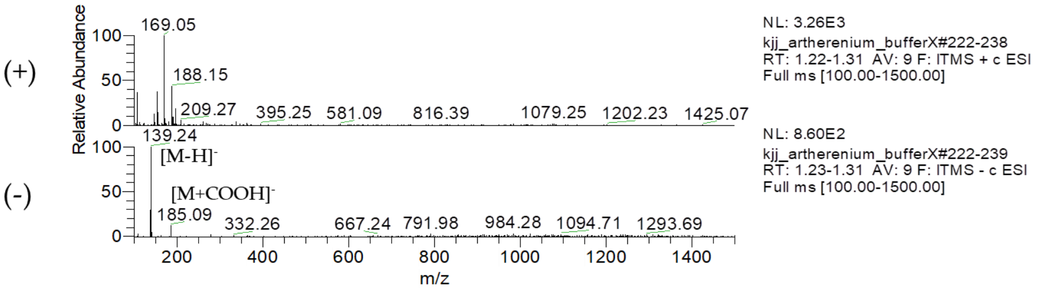

Figure S4. MS spectra of Compound **1**

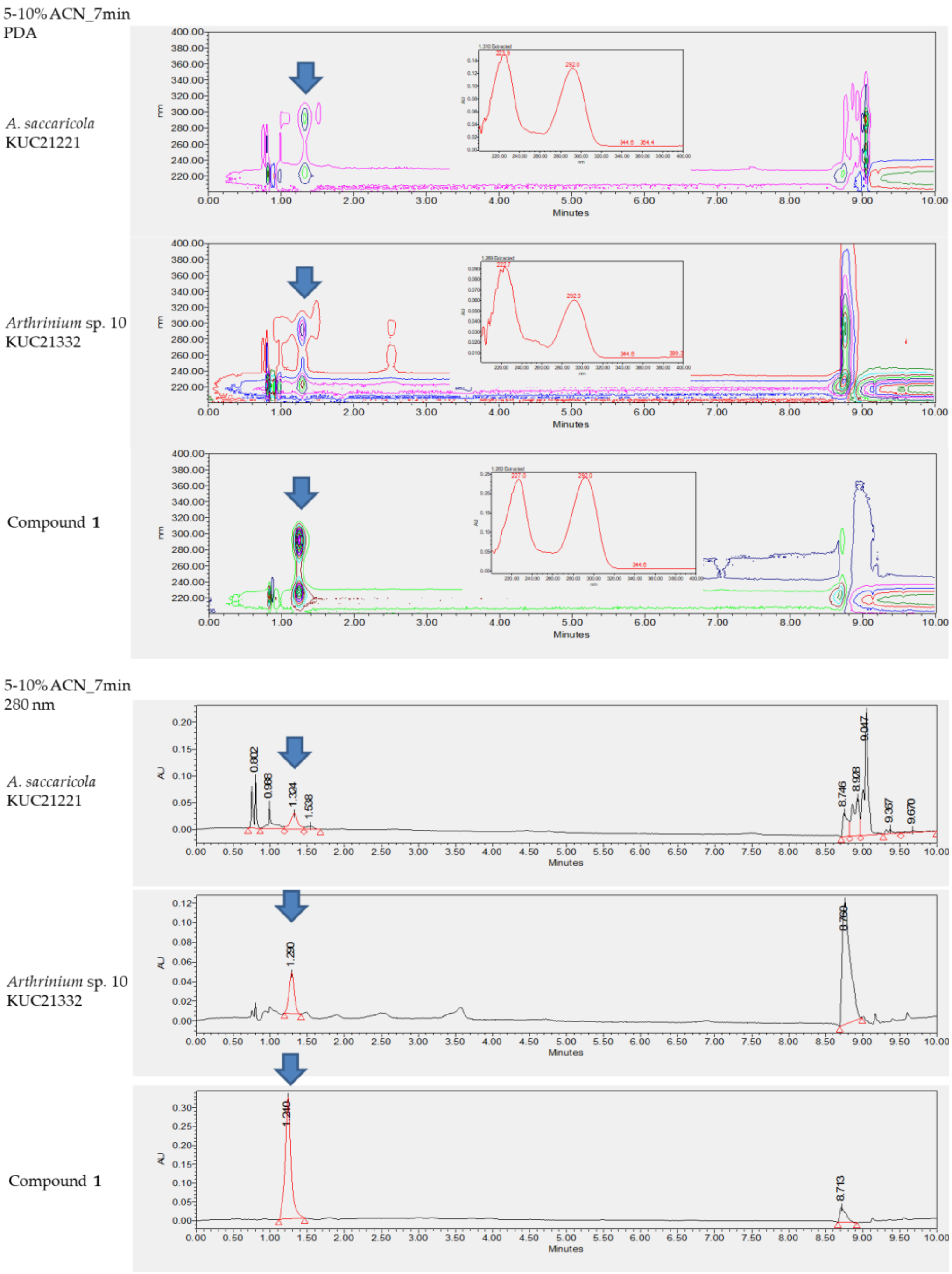

Figure S5. UPLC peaks and UV spectrum of *A. saccharicola* KUC21221 extract, *Arthrinium* sp. 10 KUC21332 extract, and Compound **1**. The arrows indicate gentisyl alcohol

Table S1. GenBank accession numbers of the reference sequences used in the phylogenetic analysis in this study.

Fungal identity	Strain ID	GenBank accession number			
		ITS	LSU	TUB	EF-1α
Arthrinium arundinis	CBS114316	KF144884	KF144928	KF144974	KF145016
A. aureum	CBS244.83	AB220251	KF144935	KF144981	KF145023
A. hydei	CBS114990	KF144890	KF144936	KF144982	KF145024
A. kogelbergense	CBS113332	KF144891	KF144937	KF144983	KF145025
	CBS113333	KF144892	KF144938	KF144984	KF145026
A. marii	CBS 113535	KF144898	KF144944	KF144990	KF145032
	CBS 114803	KF144899	KF144945	KF144991	KF145033
A. phaeospermum	CBS114314	KF144904	KF144951	KF144996	KF145038
	CBS114315	KF144905	KF144952	KF144997	KF145039
A. phragmitis	CBS135458	KF144909	KF144956	KF145001	KF145043
A. pseudosinense	CBS135459	KF144910	KF144957	N.D.	KF145044
A. pterospermum	CBS 123185	KF144912	KF144959	KF145003	N.D.
	CBS 134000	KF144913	KF144960	KF145004	KF145046
A. sacchari	CBS301.49	KF144917	KF144963	KF145006	KF145048
A. saccharicola	CBS191.73	KF144920	KF144966	KF145009	KF145051
A. xenocordella	CBS478.86	KF144925	KF144970	KF145013	KF145055
Seiridium phylicae	CPC19965	KC005787	KC005809	LT853240	LT853190

N.D. means no data.

Table S2. Marine algicolous *Arthrinium* spp. exhibiting antifungal and tyrosinase inhibition activity.

Fungal name	ID	Antifungal activity against *Asteromyces cruciatus* (MIC, μg/mL)	Tyrosinase inhibition activity (IC_{50}, μg/mL)
A. arundinis	KUC21337	N.D.	> 417
A. marii	KUC21338	N.D.	> 417
A. sacchari	KUC21340	N.D.	**104.23**
A. saccharicola	KUC21221	> 100	N.D.
	KUC21341	**100**	> 417
	KUC21342	**100**	N.D.
	KUC21343	N.D.	> 417
Arthrinium sp. 2	KUC21220	> 100	> 417
	KUC21279	N.D.	**152.07**
Arthrinium sp. 7	KUC21329	N.D.	**252.26**
Kojic acid *			49.32

N.D. means not detected. * positive control for tyrosinase inhibition activity.
